# Maternal and Placental Antibody Responses in SARS-CoV-2 Vaccination and Natural Infection During Pregnancy

**DOI:** 10.1097/INF.0000000000004704

**Published:** 2025-02-14

**Authors:** Sarah Sturrock, Breeze Cavell, Frances Alexander, Kostis Apostolakis, Camille Barro, Olwenn Daniel, Louisa Dixon, Rachel Halkerston, Tom Hall, J. Richard Hesp, Andrew M. Hill, Stephanie Leung, Suzy Lim, Nikki McStraw, Ashley Otter, Laxmee Ramkhelawon, Robert Watts, Melanie Etti, Paul Heath, Chelone Lee-Wo, Vanessa Greening, Asma Khalil, Kim Turner, Stephen Taylor, Kirsty Le Doare, Shamez Ladhani

**Affiliations:** 1 Centre for Neonatal and Paediatric Infection, St George’s, University of London, UK; 2 United Kingdom Health Security Agency, UK; 3 St George’s Hospital NHS Trust, UK; 4 St George’s, University of London, UK; 5 Keck School of Medicine, University of Southern California; 6 Makerere University-Johns Hopkins University Research Collaboration, Uganda

**Keywords:** SARS-CoV-2, antibody responses, immunogenicity, placental antibody transfer

## Abstract

**Background:**

As COVID-19 becomes endemic, understanding antibody response and transfer during pregnancy is crucial to inform policy and vaccination schedules. Whilst good immunogenicity has been shown from SARS-CoV-2 vaccines, few data are available demonstrating functional responses in pregnant populations and infants.

**Methods:**

A prospective multi-site observational study was completed across 14 centres in England from 19^th^ February 2021 21^st^ December 2022. Demographic, COVID infection and vaccination data were collected. Maternal and cord blood samples were taken at delivery, with maternal and neonatal blood samples taken at 6 weeks for participants who had been infected or vaccinated. Antibody concentrations were measured using antibody-dependent complement deposition, antibody-dependent neutrophil phagocytosis, ACE2 inhibition, and Roche and EuroImmun antibody binding assays at UK Health Security Agency.

**Results:**

Maternal vaccination and infection both produced an antibody response in 100% of mothers and 93.8% and 92.9% of neonates respectively, which persisted at 6 weeks in 95%. The strongest response was seen in mothers who were both vaccinated and infected. Anti-spike antibody response decreased almost 25-fold from first to third trimester vaccination (p=0.013). Placental transfer of antibodies post-infection showed varied results depending on the assay used, with higher transfer ratios observed in assays measuring Fc-mediated antibody effector functions and IgG-specific responses.

**Conclusion:**

Maternal vaccination is associated with good immunogenicity and successful antibody transfer to the neonate, particularly with vaccination in early pregnancy. Further study is needed to determine the mechanism by which timing of vaccination affects antibody transfer. When measuring placental transfer of antibodies, consideration of the assay to use is essential.

## Introduction

From the start of the severe acute respiratory syndrome coronavirus-2 (SARS-CoV-2) pandemic in 2019, pregnant women were identified as an at-risk group for developing severe coronavirus disease-19 (COVID-19) requiring intensive care ^1,2^. Higher rates of complications were seen in pregnant women with SARS-CoV-2 disease compared to non-pregnant populations ^3,4^. However, pregnant women were not only excluded from early vaccination and treatment studies ^5,6^, but suffered significant disruption to their antenatal care ^7^. The lack of data on which to base policy left pregnant women feeling more alone, less supported, and at a higher risk of adverse mental health outcomes ^8–11^.

Vaccination against COVID-19 promised a return to normality; however, as with many previous vaccination trials, the data regarding safety and efficacy in pregnant women trailed behind that for other groups. Although most women have now been vaccinated before becoming pregnant, understanding the quality and effectiveness of passively transferred antibodies remains vital to understand protection of the woman and her child, especially if vaccines need to be reformulated to include novel circulating variants and for safe booster dose recommendations in pregnancy.

Neonates rely on IgG, passed via the placenta to protect against infection. Studies in pandemic centers such as the USA ^12–14^ and Italy ^15,16^ have found inefficient placental transfer of SARS-CoV-2-specific IgG antibodies, suggesting that acute COVID-19 infection may compromise the process of antibody transfer^17^, particularly in third trimester infections ^18^. Suboptimal placental antibody transfer could mean that neonates remain vulnerable to SARS-CoV-2 infection in the first weeks of life. Data from the Delta and Omicron waves of the SARS-CoV-2 pandemic suggests that infection in children became more frequent and severe than earlier in the pandemic^19^, indicating that the consequences of infection may change with future mutations. As SARS-CoV-2 becomes endemic, it may mimic respiratory syncytial virus as a frequent cause of pediatric respiratory disease – therefore, understanding passive immunity is even more important from a pediatric public health perspective.

Few data describing antibody transfer during pregnancy are available for women in the United Kingdom. Additionally, few data have been used to compare the results of multiple antibody assays in the same pregnant population. It is important to understand differences in performance between assays so that future serological or vaccination studies in pregnancy can choose the most appropriate assay for their study, particularly given the difficulty of conducting vaccination trials in pregnancy. This study elected to use a mixture of anti-spike and anti-nucleocapsid antibody binding assays across different manufacturers, given the differences in performance found in previous studies, alongside several functional antibody assays^20–22^. Pregnancy is a time of significant immunomodulation, and so it is crucial to confirm whether assay performance follows similar patterns in the pregnant person as in the general population. The periCOVID study is a prospective study of pregnant women in England with and without SARS-CoV-2 infection who may or may not have been vaccinated in pregnancy aiming to understand the immunogenicity of SARS-CoV-2 vaccination and infection during pregnancy, and the protection conferred to newborns. The objectives of the study were to:

1. Quantify the immunogenicity of SARS-CoV-2 vaccination and infection during pregnancy
2. Measure transplacental transfer of anti-SARS-CoV-2 antibodies during pregnancy following maternal vaccination and maternal infection
3. Determine the short-term persistence of anti-SARS-CoV-2 antibodies transferred to the neonate
4. Explore the effect of vaccination and infection timing on maternal immunogenicity and antibody transfer
5. Compare the results of multiple anti-spike and anti-nucleocapsid functional and binding antibody assays in the same population

## Methods

### Setting

Participants were recruited at any of the periCOVID sites throughout England, including: St George’s Hospital (London), Royal Oldham Hospital, University Hospitals Dorset NHS Trust, Northwick Park Hospital (London), University Hospital Lewisham, George Eliot Hospital (Nuneaton), Chelsea and Westminster Hospital NHS Trust, Kingston Hospital, Croydon University Hospital, Worcestershire Acute Hospitals NHS Trust, Royal Bolton Hospital, Medway Maritime Hospital, Royal Free London NHS Trust, and University Hospitals Birmingham NHS Trust. Recruitment began on 23^rd^ April 2020 and closed on 21^st^ December 2022.

### Participants

Pregnant women in the United Kingdom were eligible for recruitment from 24 completed weeks of pregnancy until delivery if they were 18 years of age or older and able to provide written informed consent. Recruitment was completed via convenience sampling, with research midwifery teams contacting women known to have had a COVID-19 infection or vaccination during pregnancy as well as using local advertising through maternity services to identify interested participants. Women were excluded if they were under 18 years of age, in prison, or unable to provide written informed consent.

Participants were assigned to a study group following written, informed consent and reporting of their vaccination status and any SARS-CoV-2 PCR test taken during pregnancy or breastfeeding. The four groups were as follows:

1. Infected: at least one PCR-confirmed COVID-19 infection during pregnancy (either diagnosed via national or local screening, contact tracing, or testing due to symptoms) and no receipt of a vaccine against SARS-CoV-2 during pregnancy or breastfeeding
2. Vaccinated: received a vaccine against SARS-CoV-2 during pregnancy or breastfeeding, with no positive PCR test during pregnancy or breastfeeding
3. Both: received a vaccine against SARS-CoV-2 and had at least one positive PCR test for SARS-CoV-2 during pregnancy or breastfeeding
4. Neither: no positive PCR test for SARS-CoV-2 and no receipt of a vaccine against SARS-CoV-2 during pregnancy or breastfeeding

If a participant subsequently had additional PCR test results or vaccinations during the study period, their group was adjusted accordingly (for example, a participant in the Infected group who then had a vaccination would move to the Both group).

### Data and sample collection

Demographic and pregnancy information (including medical comorbidities, BMI, any pregnancy-related complications) was collected at recruitment. Additional demographic fields, such as ethnicity, were added partway through the study via a protocol amendment as associations between SARS-CoV-2 disease severity and ethnicity became apparent. These data were added retrospectively for participants wherever possible. Study data (demographic and clinical) was collected via REDCap, a secure online research database. Participants in the exposed group were divided into subgroups according to the dates of major COVID-19 waves in the United Kingdom^23^: Wave 1 (1^st^ January 2020 to 4^th^ July 2020), Wave 2 (5^th^ July 2020 to 19^th^ May 2021), Wave 3 (20^th^ May 2021 to 5^th^ June 2022) and Wave 4 (6^th^ June 2022-present). Need for oxygen therapy and admission to intensive care were both recorded as markers of disease severity.

At the point of delivery, maternal peripheral blood, placental swabs, and umbilical cord blood were obtained. Perinatal information including method of delivery, birthweight, and delivery complications were recorded. Within 48 hours of delivery, a breast milk swab was obtained. For the exposed and vaccinated groups, a further maternal blood sample, neonatal blood sample, and breast milk sample (where available) were collected at 6 weeks postpartum. Follow up information for this group including any hospital attendances or admissions, confirmed SARS-CoV-2 infection, or vaccination against SARS-CoV-2, was recorded at 6 weeks (and up to 10 weeks) postpartum.

### Laboratory analyses

All laboratory analyses were undertaken at UK Health Security Agency, Porton Down.

### Serum bactericidal assay

In addition to the SARS-CoV-2 assays described below, functional antibody responses to Bordetella pertussis were also measured on a subset of samples using a serum bactericidal assay (SBA)^24^. This assay was chosen because pertussis vaccination is routinely offered in pregnancy in the UK^25^, allowing comparison of functional activity against COVID-19 to the immunogenicity of the pertussis vaccination and the placental transfer of resultant antibodies.

The following assays were conducted to measure response to SARS-CoV-2:

- Antibody-dependent complement deposition (ADCD) assay
- Antibody-dependent neutrophil phagocytosis (ADNP) assay
- Antibody and complement-dependent ACE2 inhibition (ACDA2I) assay
- Antibody binding
- Microneutralization assay (MNA)

Laboratory methods for these assays can be found in Methods, Supplemental Digital Content 1.

### Statistical methods

The study was powered to estimate the overall seroprevalence in the population of pregnant women in the UK. Recruitment of 1000 pregnant women allowed good precision of the estimation of overall seroprevalence if the observed proportion of seropositive women was as low as 5% using a 95% confidence interval (3.7%-6.5%). However, the study was halted before the original recruitment target of unvaccinated women was met as vaccination was introduced during the study period and sites were no longer able to recruit the intended numbers of uninfected and unvaccinated participants.

Seropositivity is described per 100,000 maternities or 1000 livebirths as appropriate with 95% confidence intervals. Perinatal outcomes were compared between groups using Kruskal-Wallis tests. Normality of antibody assay results was verified using a Shapiro Wilks test, and comparisons made between groups using Kruskal Wallis tests. All univariate plots were visualized and analyzed using Graphpad software, version 9.0 for Windows (San Diego, California USA). Transfer ratios were calculated by dividing neonatal antibody by maternal antibody result. For meaningful ratios, participants were excluded from transfer ratio calculation where either of the mother-infant pair tested below the reportable range of the assay, or for the Euroimmun Anti-SARS-CoV-2 QuantiVac ELISA (IgG), above the reportable range. Statistical analysis was performed using R Studio version 4.2.2^26^. Simple and multiple linear regression analysis was completed for maternal delivery antibody results, log transformed, to examine the effect of study group and maternal age, ethnicity, and BMI.

### Ethical approval

The study protocol was reviewed by UK Health Security Agency. Ethical approval was granted by the Newcastle and North Tyneside 1 Research Ethics Committee (REC reference 20/NE/0284).

## Results

### Participants

A total of 881 participants were recruited to the periCOVID study. Participants were assigned to final groups as follows (see Table, Supplemental Digital Content 2): 173 had a positive PCR test for SARS-CoV-2 during pregnancy (‘Infected’), 43 received a vaccine against SARS-CoV-2 (‘Vaccinated’), 38 had both received a vaccine and had a positive PCR test (‘Both’), and 627 had not been vaccinated or had a positive PCR test (‘Neither’). Any available serological data from included recruited participants were used for maternal transfer assessment and so all participants were included in the final analysis. One maternal placenta and one baby nasal swab tested positive for SARS-CoV-2 at delivery via PCR, but delivery room cross-contamination could not be excluded in these cases.

The median age of included participants was 32 years (IQR 28-32), and 72% of participants identified as White. See Table, Supplemental Digital Content 2 for demographic features of participants. 250 (29%) of included participants had at least one medical comorbidity, and 307 (35%) experienced at least one pregnancy complication. Most participants in the Infected or Both groups had experienced symptoms of COVID-19 (n=178, 84%). However, few of these participants required oxygen because of their disease (n=7, 3%).

### Seropositivity and antibody response

Amongst participants who were uninfected and unvaccinated (‘Neither’), 126 (14%) participants tested positive for antibodies against SARS-CoV-2 in at least one assay at delivery, giving a group seropositivity of 14,302 per 100,000 maternities. All Vaccinated participants with available serology results were seropositive, as were those in the Infected and Both groups.

Seropositivity in all neonates with serology results available at delivery was 458 per 1000 live births. The highest neonatal seropositivity was 938 per 1000 live births in the Vaccinated group, followed by 929 per 1000 in the Infected group, and 893 in the Both group (although the Both groups had the smallest available sample of results, n=28). Seropositivity in the group of neonates whose mothers had not been infected or received a vaccine was 267 per 1000 live births.

There were 190 participants in whom the date of COVID-19 diagnosis was known, and therefore for whom a Wave for their infection could be assigned. Of these, 2 were in Wave 1, 122 in Wave 2, and 66 in Wave 3. All these participants where serology was known were seropositive.

Participants who had been infected with SARS-CoV-2 had the highest antibody responses against the N protein (p<0.001, see Figure and Table, Supplemental Digital Content 3 and 4, respectively). In contrast, the Both group showed the highest antibody responses in the S protein assays, followed by Vaccinated (p<0.001, see Figure and Table, Supplemental Digital Content 3 and 4, respectively). Participants infected in the third trimester generally showed higher antibody results across all assays compared to those infected earlier in pregnancy, although this trend did not reach statistical significance in the two Roche assays (See Table, Supplemental Digital Content 4). Conversely, those vaccinated in the 1^st^ trimester showed a higher antibody response than those vaccinated later in pregnancy across all assays. However, results varied by assay and differences by vaccination timing only reached statistical significance in the Roche S assay.

A similar pattern was seen amongst neonatal participants; the N protein assays showed the strongest responses amongst Infected participants, whereas the S protein assays showed a stronger response in the Both group (p<0.001, see Figure and Table, Supplemental Digital Content 5 and 6, respectively). A mixed picture was seen in neonatal results at delivery by trimester of infection, but the only statistically significant result was an increase in the result of the ADCD N assay with later infection in pregnancy. A consistent trend was seen with the neonates of participants vaccinated in the 1^st^ trimester having a stronger response than those born to participants vaccinated later in pregnancy (See Table, Supplemental Digital Content 6).

Multiple linear regression analysis of log-transformed maternal delivery antibody results showed that study group was by far the strongest predictor of antibody response. Maternal age and BMI did not show any statistically significant effect on antibody response, although Black ethnicity did show a positive relationship with antibody levels in both the Roche assays, but not in any of the other assays (see Tables, Supplemental Digital Content 7-11).

### Placental transfer

Increased levels of functional antibodies against the spike (S) and nucleocapsid (N) of SARS-COV-2, as measured by the ADCD S and N, ADNP and ACDA2I, were observed in the cord compared to maternal blood in Infected mother:cord dyads (see Table, Supplemental Digital Content 12). A similar pattern was observed in the Vaccinated and Both groups, although with lower numbers of participants eligible for inclusion in transfer ratio analysis. This observation is consistent with what is seen with functional antibodies against *B. pertussis* measured within the same cohort (see Figure, Supplemental Digital Content 13). Higher levels of spike-specific IgG as shown by the Euroimmun ELISA were also observed in the cord compared to maternal blood, as were antibodies against nucleocapsid in the Roche N ECLIA. Conversely, low transfer ratios were seen in the Roche S ECLIA and MNA and these were significantly lower than all the other antibody responses measured. Only the Roche ECLIA showed increased placental transfer of anti-nucleocapsid antibodies compared to anti-spike antibodies. (see Figure, Supplemental Digital Content 13).

### Antibody persistence

All pregnant participants who had positive antibodies at delivery and had results available at 6 weeks post-delivery still had a positive result in at least one assay. Ninety-five percent of neonatal participants born to participants with positive antibodies at delivery with 6 week results available remained seropositive. Two neonates who were seronegative at delivery had a positive antibody result at 6 weeks. All 4 neonates who had become seronegative at 6 weeks were in the Infected group.

## Discussion

In this large seroepidemiology study of COVID-19 in pregnant women in England, we found placental transfer of antibodies against SARS-CoV-2 occurred after natural infection, and with higher levels of anti-S antibody after experiencing both infection and vaccination. We found that vaccination in the first trimester gave the highest antibody concentrations at delivery, although results varied by different assays.

In agreement with other studies internationally, we found that anti-spike antibody response increased with increasing time between vaccination and delivery^27^. Studies of natural SARS-CoV-2 infection during pregnancy have found placental antibody transfer is proportional to the time between infection and delivery^28^. All participants who had been infected, vaccinated or both with available results were seropositive at delivery. The seropositivity in our cohort is similar to that in other pregnant cohorts in North America^29,30^ and global general adult cohorts^31,32^, although it should be noted that our sample was not random and therefore cannot be used to infer the seroprevalence amongst UK pregnant people. Our finding that many seropositive women had not been aware of a previous infection is in keeping with other seroepidemiologic studies in pregnancy in the UK^33^.

We also found reliable transfer of maternal antibodies against SARS-CoV-2 to infants in this study in mothers infected and vaccinated. Studies in Israel, Italy and the USA found successful IgG transfer from maternal blood to cord blood and breastmilk, particularly following vaccination^27,34,35^. Maternal IgG antibodies are transferred to the neonate across the placenta, dependent on neonatal Fc receptors (FcRn) within the placenta^36^. Previous studies suggest that the de novo infection with SARS-CoV-2 and resultant inflammation alters Fc glycosylation, affecting IgG transfer, although these changes normalize over time^18^. This could explain why more neonatal antibodies were found in neonates born to mothers infected later in pregnancy, and why we found placental transfer ratios >1 in some of the assays used in this study having included participants infected in all stages of pregnancy.

Our results for transplacental transfer ratios varied across the different assays used, with lower transfer ratios seen in the Roche S ECLIA and MNA assays. The lower placental transfer ratio observed in the Roche S assay is likely due to the assay measuring total antibodies against spike, only some of which would be transferred. However, it is interesting that the Roche N assay, which similarly measures total antibodies but against nucleoprotein had a significantly better placental transfer ratio. Although not significant, the placental transfer ratio seen with the ADCD N assay was also higher than that observed in the ADCD S assay. These results suggest that IgG antibodies against SARS-CoV-2 nucleoprotein may be preferentially transferred across the placenta compared to IgG antibodies against SARS-CoV-2 spike. As transplacental transfer of IgG has been shown to be dependent not only on IgG subclass but also Fc glycosylation state^17,18,37^, this would imply that SARS-CoV-2 infection often leads to the generation of more Fc-effector functional IgG to nucleoprotein than spike. While the sample size of mother-cord dyads with MNA assay results was smaller compared to other functional assays, the difference in transfer ratios could be attributed to the selective placental transfer of antibodies that bind more effectively to FcRn. The neutralizing function of antibodies is achieved by binding to specific epitopes on pathogens, blocking their ability to infect host cells. This mechanism involves the Fab region and is not restricted to specific antibody isotypes or subclasses, thus encompassing more than just IgG. In contrast, complement-dependent Fc-mediated antibody effector functions, as measured by antibody-dependent complement deposition (ADCD) and antibody-dependent neutrophil phagocytosis (ADNP) assays, are more IgG driven and likely have optimized Fc regions for stronger FcRn interactions. This optimization could enhance the efficiency of Fc-mediated antibody transfer across the placenta, resulting in the higher transfer ratios observed in assays measuring Fc-mediated antibody effector functions. Of note is the positive transfer ratio seen using the ACDA2I, which can be seen as a more functional surrogate of the MNA, as the deposition of complement relies on the Fc-effector function of the bound antibody.

We found the highest anti-spike antibody response in those who had been infected and vaccinated as opposed to infected or vaccinated alone, with vaccination also showing a stronger response than natural infection. This is in agreement with other studies of pregnant women^38–40^. The relationship between timing of vaccination and consequent antibody levels in the mother and the infant is dependent on multiple factors. Optimal vaccination timing for antibody transfer appears to depend on the type of vaccine given, with mRNA-1273 vaccine in particular showing a clear relationship between timing of vaccine and maternal response^41^. Given the small numbers of vaccinated women in this study, we were not able to analyze whether the type of vaccine affected antibody transfer.

We found that first trimester vaccination led to higher antibody levels in infants, which could relate to better maternal immunogenicity in the first trimester. Vaccination in the second trimester has been associated with reduced maternal immunogenicity, which may be due to the necessary changes in the maternal immune system to promote tolerance to the foetus^41^. First trimester vaccination has been associated with lower umbilical antibody titers, despite more efficient transplacental transfer, which may be due to waning maternal antibody titers over the rest of the pregnancy. We did not find this in our study, and we hypothesize that this is due to the fact that FcRN saturation had not yet been reached, and is more likely to occur later in pregnancy^42^.

Lower transfer ratios for anti-SARS-CoV-2 antibodies have been found following third trimester infections compared to second trimester infections in other studies, in contrast to our results^14,18,27,39,43^. This could be due to different SARS-CoV-2 variants causing different inflammatory changes in the placenta, and thus affecting transplacental transfer^44^.

Antibodies transferred to the infants in this study persisted with almost all infants seropositive at delivery testing positive at 6 weeks. Although we found a stronger anti-spike antibody response in mothers who had been vaccinated compared to those with a natural infection, there was no significant difference between study groups in the persistence of the infant antibodies at 6 weeks post-delivery. Further work could investigate antibody transfer and persistence in infants whose mothers had completed a full course of COVID-19 vaccination including boosting, or who had been vaccinated in the first or second trimester of infection to determine an optimal schedule.

The strength of this study lies in its collection of data and serologic samples from women and neonates across the UK, where seroepidemiologic study of SARS-CoV-2 during pregnancy has been limited compared to other pandemic centers. We were also able to capture data across the principal waves of COVID-19 in England, including at least 2 predominant variants. These factors both contribute to the generalizability of the study to most UK maternity populations. Additionally, by including both delivery and 6-week samples, we were able to study the persistence of serologic protection against SARS-CoV-2 – a key point of parental anxiety during and after the pandemic. Finally, this study can compare results for multiple assays, highlighting key differences in their findings from the same population. This should be useful for future maternal vaccination studies to inform assay choice.

The main limitation of this study was the incompleteness of data resulting from loss to follow up and incomplete sample sets, restricting our ability to calculate transfer ratios for the whole sample for all assays. The pandemic and lockdown restrictions posed unique challenges to completing follow up visits and sample collection. The study did not use random sampling, and therefore we cannot use this data to infer the prevalence of COVID-19 infection or antibody response within the UK pregnant population. Some demographic factors, such as ethnicity, are unbalanced between study groups, likely due to the convenience sampling method and the differential likelihood of different groups to have received a vaccine or PCR test. We have tried to limit the effect of this imbalance by including ethnicity in regression analyses. Additionally, the study was halted before reaching the original recruitment targets due to a lack of unvaccinated and unexposed women in the community as the COVID-19 pandemic entered its second year in the UK. Finally, although our cohort displayed a wide range in terms of their time from exposure or vaccination to delivery, their ages, and their ethnicities, there was less variation in the clinical presentation and severity of their COVID-19 disease than seen overall in the UK. This limited our ability to ascertain the associations between symptoms or severity and maternal antibody response or transfer.

## Conclusion

We found strong evidence of transplacental transfer of antibodies against SARS-CoV-2 with higher antibody levels in mother and baby with earlier vaccination during pregnancy. Our use of functional antibody assays looking at Fc-mediated antibody effector functions to measure seropositivity supports vaccination against SARS-CoV-2 in pregnancy as a protective measure for mother and baby. It also serves as a cautionary tale for future pandemics, when we must carefully consider which antibody assays are used prior to conducting large seroepidemiology or vaccine studies.

## Supplementary Material

Edited final abstract

SDC 1

SDC 2

SDC 3

SDC 4

SDC 5

SDC 6

SDC 7

SDC 12

SDC 13

SDC 8-11
