# Supplementary material for: Maternal and Placental Antibody Responses in SARS-CoV-2 Vaccination and Natural Infection During Pregnancy
Source: Pediatr Infect Dis J. 2025 Feb 14;44(2):S32–7. doi: 10.1097/INF.0000000000004704 (PMC7617455; doi:10.1097/INF.0000000000004704)
Supplement: Supplementary file 1 [file inf-44-s032-s001.pdf]

SUPPLEMENTAL DIGITAL CONTENT 1. Methods

Antibody-dependent complement deposition (ADCD) assay

SPHERO carboxyl magnetic blue-fluorescent beads of varying intensities (Spherotech, USA) were coupled with SARS-CoV-2 whole spike protein (Lake Pharma, 46328) and recombinant nucleoprotein prepared in-house using a two-step sulpho-NHS/EDC process44. To prepare the nucleoprotein, the full-length SARS-CoV-2 nucleocapsid gene was optimised for expression in human cells and synthesised and subcloned into pcDNA3.1 expression vector (GeneArt, ThermoFisher) for transient expression in Expi293 cells. The nucleoprotein was expressed with a C-terminal His(6) tag and purified from the cytosolic fraction after detergent lysis and sonication using a 5 ml HisTrap excel column (as per manufacturer's instructions, Cytiva). The eluted pool was dialysed against 50 mM HEPES, NaOH, pH8.0, before being applied to a 5 ml SP-Sepharose HiTrap column. Following elution with 50 mM HEPES, NaOH, pH8.0 + 1M NaCl (gradient elution, 20 column volumes) purified nucleoprotein was dialysed against 50 mM HEPES-NaOH, pH8.0, 250 mM NaCl, 1 mM EDTA, 20% (w/v) sucrose and stored at -80°C. Heat-inactivated NIBSC Anti-SARS-CoV-2 Antibody Diagnostic Calibrant (NIBSC, 20/162) at an initial 1:40 dilution in blocking buffer (BB; PBS, 2% BSA) was added in duplicate to a V-bottom 96-well microtiter plate and serially diluted 2:3 in 20 µL BB. Heat-inactivated test serum (3 µL in duplicate) were added to 27 µL BB and serially diluted 1:3 in 20 µL BB. This was followed by 20 µL of SARS-CoV-2 spike and nucleocapsid protein-coated magnetic beads (50 beads per µL for each bead type) to give a multiplexed assay with a final 1:3 serial dilution range starting at 1:20. The serial dilution for NIBSC 20/162 standard started at 1:80. The mixture was incubated at 25°C for 30 min with shaking (900rpm). The beads were washed twice in 200 µL wash buffer (WB; BB+0.05% Tween-20), then resuspended in 50µL BB containing 12.5% IgG- and IgM-depleted human plasma45 and incubated at 37°C for 15 min with shaking (900rpm). Beads were next washed twice with 200 µL WB and resuspended

in 100 µL FITC-conjugated rabbit anti-human C3c polyclonal antibody (Abcam) diluted 1:500 in BB and incubated in the dark at 25°C for 20 min. After two more washes with 200 µL WB, the samples were resuspended in 40 µL HBSS and analysed using an iQue Screener Plus® with iQue Forecyt® Standard Edition 9.0 (R3) software (Sartorius, Germany). For each sample, a minimum of 50 beads (per bead type) were collected. Conjugated beads were gated based on forward scatter and side scatter and then spike-conjugated and nucleocapsid-conjugated beads separated and gated by fluorescence in the BL4 channel (488 nm – 675/30 nm). The fluorescent-bead population was gated and measured for FITC Median Fluorescent Intensity (MFI), which represents deposition of C3b/iC3b. The NIBSC 20/162 calibrant was assigned an arbitrarily unitage of 1,000 complement activating units (CAU)/mL and plotted as a 4PL curve with 1/Y2 weighting and the linear range calculated. The mean MFI from each sample was interpolated against the NIBSC 20/162 4PL curve and based on the concentration that hit the linear range was multiplied by the dilution factor to assign activity of the sera as CAU.

Antibody-dependent neutrophil phagocytosis (ADNP) assay

The ADNP protocol was adapted from previous studies46. 1 µm carboxylate-modified crimson FluoSpheres™ beads (Thermo Fisher, USA) were coupled with SARS-CoV-2 whole spike protein (Lake Pharma, 46328) using a two-step sulpho-NHS/EDC process [25]. Heat-inactivated NIBSC Anti-SARS-CoV-2 Antibody Diagnostic Calibrant (NIBSC, 20/162) at an initial 1:85 dilution in DPBS-GACM buffer (Dulbecco's PBS supplemented with 5.5 mM glucose, 0.5% w/v BSA, 0.9 mM CaCl2 and 0.5 mM MgSO4 at pH 7.4) was added to a V-bottom 96-well microtiter plate and serially diluted 5:6 in 20 µL DPBS-GACM. Heat-inactivated test sera (5 µL) were added to 20 µL DPBS-GACM and serially diluted 1:2 in 12 µL DPBS-GACM. 5 µL from the calibrant and the test sera serial dilutions were transferred in duplicate to a U-bottom 96-well microtiter plate, followed by the addition of 5 µL spike-

conjugated beads (4×10^4 beads/µL). The serial dilution for NIBSC 20/162 standard started at 1:170, and for test samples started at 1:10. The mixture was incubated at 37°C for 1h with shaking (900rpm). Next, 5 µL of IgG- and IgM-depleted human plasma45 diluted 1/10 in DPBS-GACM was added to the mix as an exogenous complement source and this was immediately followed by the addition of 10 µL of granulocyte-differentiated HL-60 cells (ATTC, CCL-240; differentiated with 0.8% N,N-dimethylformamide for 5 days) resuspended in DPBS-GACM at a concentration of 2×10^6 cells/mL. The plate was incubated at 37°C for a further 1h with shaking (900rpm). At the end, phagocytosis was stopped by placing the plate on ice and adding 20µL of cold DPBS with 0.02% w/v EDTA in every well. Samples were analysed using an iQue Screener Plus® with iQue Forecyt® Standard Edition 9.0 (R3) software (Sartorius, Germany). Phagocytising cells were gated based on forward scatter and side scatter and MFI in the BL4 channel (488 nm – 675/30 nm) measured, which represents uptake of fluorescent beads. The NIBSC 20/162 calibrant was arbitrarily designated an opsonophagocytic activity equal to 1,000 phagocytosis units (PU)/mL and plotted as a 4PL curve with 1/Y2 weighting and the linear range calculated. The mean MFI from each sample was interpolated against the NIBSC 20/162 4PL curve and based on the concentration that hit the linear range was multiplied by the dilution factor to assign activity of the sera as Interpolated Phagocytosis Units (IPU).

Antibody and complement-dependent ACE2 inhibition (ACDA2I) assay

SPHERO carboxyl magnetic blue-fluorescent beads were coupled with SARS-CoV-2 whole spike protein as above. Heat-inactivated NIBSC 20/162 at an initial 1:25 dilution in BB was added in duplicate to a V-bottom 96-well microtiter plate and serially diluted 2:3 in 20 µL BB. Heat-inactivated test serum (12 µL in duplicate) were added to 48µL BB and serially diluted 2:3 in 20 µL BB. This was followed by 20 µL of SARS-CoV-2 spike protein-coated magnetic

beads (50 beads per µL) to give a final 2:3 serial dilution range starting at 1:10. The serial dilution for NIBSC 20/162 standard started at 1:50. The mixture was incubated at 25°C for 30 min with shaking (900rpm). The beads were washed twice in 200µL wash buffer (WB; BB+0.05% Tween-20), then resuspended in 50 µL BB containing 10% IgG- and IgM-depleted human plasma45 and incubated at 37°C for 15 min with shaking (900rpm). Beads were next washed twice with 200 µL WB and resuspended in 50 µL Human ACE2 (18-615) Recombinant Protein, Sheep FC-Tag (The Native Antigen Company Limited, REC31876) at 1.0 µg/mL concentration in BB and incubated at 37°C for 30 min with shaking (900rpm). After two more washes with 200 µL WB, the samples were resuspended in 100 µL FITC-conjugated AffiniPure Rabbit Anti-Sheep Polyclonal IgG, Fc-Fragment Specific (Jackson ImmunoResearch) at 1:500 dilution for 20 min in the dark at 25°C with shaking (400rpm). Following two final washes with 200 µL WB, the samples were resuspended in 20 µL HBSS and analysed using an iQue Screener Plus® with iQue Forecyt® Standard Edition 9.0 (R3) software as above. The fluorescent-bead population was gated and measured for FITC MFI, which quantifies inhibition of Spike-ACE2 interaction due to the formation of antibody-complement immune complexes. The NIBSC 20/162 calibrant was assigned an arbitrarily unitage of 1,000 ACE-2 inhibitory units (AIU)/mL and plotted as a 4PL curve with 1/Y2 weighting and the linear range calculated. The mean MFI from each sample was interpolated against the NIBSC 20/162 4PL curve and based on the concentration that hit the linear range was multiplied by the dilution factor to assign activity of the sera as AIU.

Antibody binding

SARS-CoV-2 antibody testing was performed using commercial platforms. Roche Elecsys Anti-SARS-CoV-2 S (Roche S) and Anti-SARS-CoV-2 (Roche N) electrochemiluminescence immunoassay (ECLIA) which measures total antibodies to the spike protein RBD or

nucleocapsid protein47,48. As well as EuroImmun Anti-SARS-CoV-2 QuantiVac enzyme-linked immunosorbent assay (ELISA) (IgG) targeting the S1 antigen[28]. While the Roche S and EuroImmun assays are either quantitative or semi-quantitative, reporting results as units (U)/mL and relative units (RU)/mL respectively, the Roche N assay is primarily qualitative. Nonetheless, the signal/cut-off (S/CO) ratio used in the Roche N assay provides a relative measure of antibody levels within the sample by comparing the signal generated by the sample to that of an internal reactive calibrator.

Microneutralization assay (MNA)

MNAs were performed on live virus SARS-CoV-2/human/AUS/VIC01/2020 [Victoria] strain and neutralisation titres giving 50% focus reduction determined 49.
