# Supplementary material for: Maternal and Placental Antibody Responses in SARS-CoV-2 Vaccination and Natural Infection During Pregnancy
Source: Pediatr Infect Dis J. 2025 Feb 14;44(2):S32–7. doi: 10.1097/INF.0000000000004704 (PMC7617455; doi:10.1097/INF.0000000000004704)
Supplement: Supplementary file 2 [file inf-44-s032-s002.pdf]

SUPPLEMENTAL DIGITAL CONTENT 2. Characteristics of participants

		All (n=881)	Infected (n=173)	Vaccinated (n=43)	Both (n=38)	Neither (n=627)
Age (years)	Median	32.0	33.0	34.0	33.5	31.0
	IQR	28.0-32.0	29.0-36.0	30.5-37.0	31.3-36.8	27.0-35.0
BMI (kg/m^2)	Median	27.0	27.0	24.5	25.6	27.0
	IQR	23.0-31.6	23.0-33.1	23.0-27.0	23.0-28.2	23.0-31.7
Ethnicity N (%)	White	637 (72%)	85 (49%)	32 (74%)	24 (63%)	496 (79%)
	Asian/Asian British	75 (9%)	9 (5%)	8 (19%)	4 (11%)	54 (9%)
	Black/African/Caribbean/British	24 (3%)	4 (2%)	0 (0%)	0 (0%)	20 (3%)
	Other	23 (3%)	8 (5%)	2 (5%)	1 (3%)	12 (2%)
	Mixed/Multiple	13 (1%)	1 (1%)	0 (0%)	0 (0%)	12 (2%)
	Not stated	109 (12%)	66 (38%)	1 (2%)	9 (24%)	33 (5%)
