# Supplementary material for: Maternal and Placental Antibody Responses in SARS-CoV-2 Vaccination and Natural Infection During Pregnancy
Source: Pediatr Infect Dis J. 2025 Feb 14;44(2):S32–7. doi: 10.1097/INF.0000000000004704 (PMC7617455; doi:10.1097/INF.0000000000004704)
Supplement: Supplementary file 4 [file inf-44-s032-s004.pdf]

SUPPLEMENTAL DIGITAL CONTENT 4. Maternal antibody results at delivery (geometric mean)

		Maternal antibody results at delivery				
		ADCD N (CAU/mL)	ADCD S (CAU/mL)	Roche N (S/CO ratio)	Roche S (U/mL)	Euroimmun (RU/mL)
Study group	Infected	82.2	37.2	12.5	62.2	1.7
	Vaccinated	18.9	80.3	0.1	278.4	4.8
	Both	61.0	590.5	3.9	6523.5	15.5
	Neither	18.2	7.2	0.2	1.5	0.2
	p value*	<0.001	<0.001	<0.001	<0.001	<0.001
Trimester of infection	First	33.0	42.2	8.9	221.2	2.0
	Second	60.8	45.1	13.7	127.3	1.7
	Third	145.9	125.8	11.0	276.6	2.4
	p value*	0.001	0.024	0.734	0.372	0.041
Trimester of vaccination	First	43.4	349.3	3.7	5005.6	3.1
	Second	27.9	262.3	0.3	3284.6	11.1
	Third	30.5	95.3	0.4	207.1	4.2
	p value*	0.129	0.055	0.155	0.013	0.107

*p value for Kruskal Wallis test
