# Supplementary material for: Maternal and Placental Antibody Responses in SARS-CoV-2 Vaccination and Natural Infection During Pregnancy
Source: Pediatr Infect Dis J. 2025 Feb 14;44(2):S32–7. doi: 10.1097/INF.0000000000004704 (PMC7617455; doi:10.1097/INF.0000000000004704)

SUPPLEMENTAL DIGITAL CONTENT 5. Neonatal antibody results at delivery (cord) for a) antibody-dependent complement deposition assay against spike protein, b) antibody-dependent complement deposition assay against nucleocapsid protein, c) antibody-dependant neutrophil phagocytosis, d) antibody and complement-dependant ACE2 inhibition assay, e) Roche total antibody binding to RBD, f) Roche total antibody binding to nucleocapsid protein, g) Euroimmun IgG binding to S1 antigen. Geometric mean with 95% CI plotted.

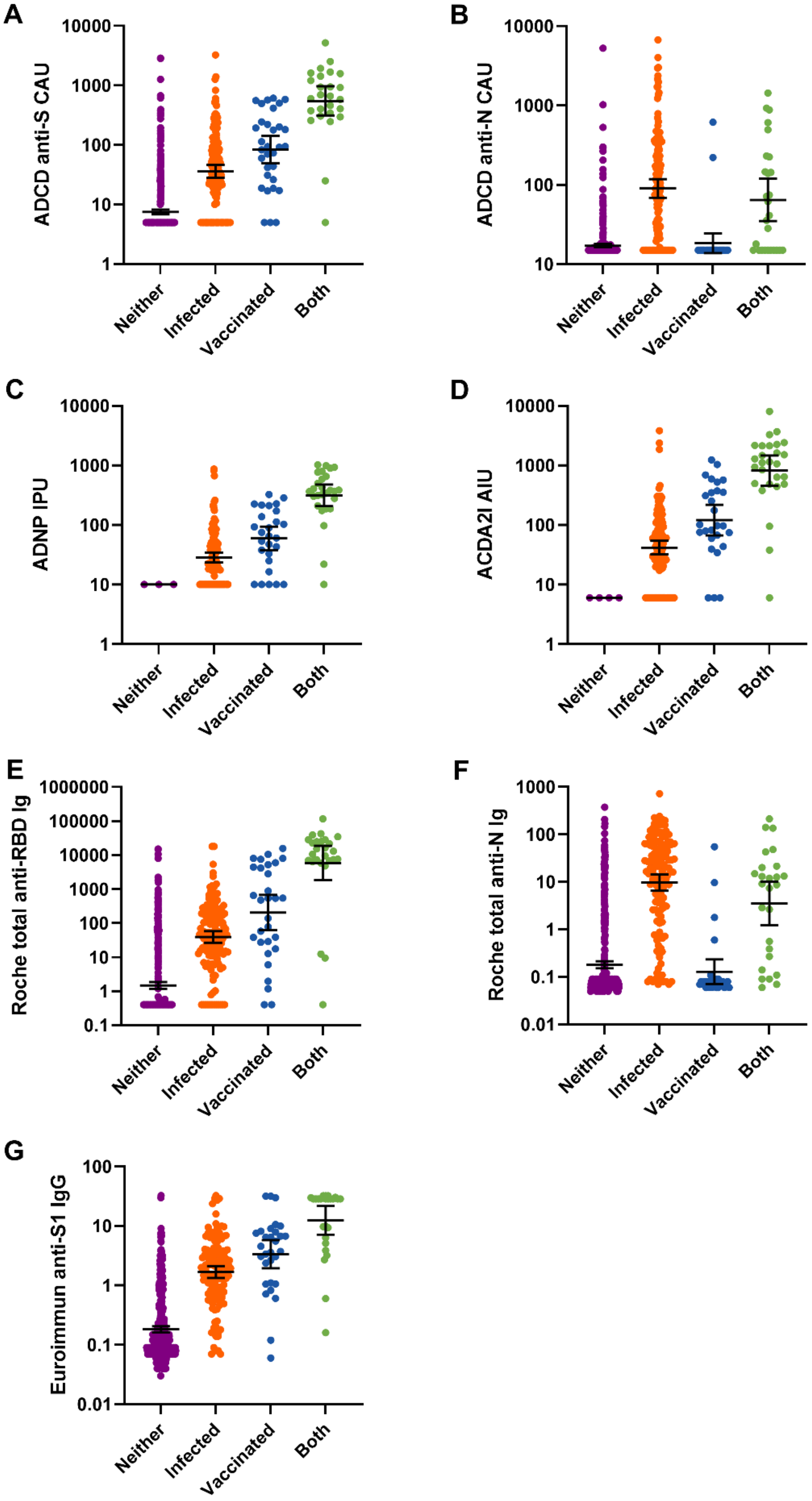

Supplement: Supplementary file 5 [file inf-44-s032-s005.pdf]
