# Supplementary material for: Maternal and Placental Antibody Responses in SARS-CoV-2 Vaccination and Natural Infection During Pregnancy
Source: Pediatr Infect Dis J. 2025 Feb 14;44(2):S32–7. doi: 10.1097/INF.0000000000004704 (PMC7617455; doi:10.1097/INF.0000000000004704)
Supplement: Supplementary file 6 [file inf-44-s032-s006.pdf]

SUPPLEMENTAL DIGITAL CONTENT 6. Neonatal antibody results at delivery (geometric mean)

		ADCD N (CAU/mL)	ADCD S (CAU/mL)	Roche N (S/CO ratio)	Roche S (U/mL)	Euroimmun (RU/mL)
Study group	Infected	89.0	35.9	9.9	39.0	1.7
	Vaccinated	18.3	83.9	0.1	203.8	3.3
	Both	64.6	549.0	3.5	5831.8	12.4
	Neither	17.1	7.5	0.2	1.5	0.2
	p value*	<0.001	<0.001	<0.001	<0.001	<0.001
Trimester of infection	First	28.5	57.9	12.8	270.3	1.9
	Second	75.0	54.7	18.2	103.5	2.3
	Third	124.3	88.8	5.4	128.4	1.9
	p value*	0.001	0.470	0.217	0.269	0.464
Trimester of vaccination	First	58.9	387.5	4.3	6873.9	5.0
	Second	24.9	343.5	0.4	8775.0	11.7
	Third	26.8	62.6	0.4	89.7	3.6
	p value*	0.565	0.006	0.645	0.002	0.044

*p value for Kruskal Wallis test
