# Supplementary material for: Maternal and Placental Antibody Responses in SARS-CoV-2 Vaccination and Natural Infection During Pregnancy
Source: Pediatr Infect Dis J. 2025 Feb 14;44(2):S32–7. doi: 10.1097/INF.0000000000004704 (PMC7617455; doi:10.1097/INF.0000000000004704)
Supplement: Supplementary file 7 [file inf-44-s032-s007.pdf]

SUPPLEMENTAL DIGITAL CONTENT 7. Simple and multiple linear regression results for log-transformed maternal Roche N assay results at delivery

Explanatory variables		Simple linear regression			Multiple linear regression		
		Estimate	95% CI	p value	Estimate	95% CI	p value
Study group*	Infected	1.850	1.69, 2.00	<0.001	1.840	1.65, 2.04	<0.001
	Vaccinated	-0.180	-0.46, 0.10	0.204	-0.170	-0.45, 0.10	0.219
	Both	1.380	1.09, 1.68	<0.001	1.440	1.12, 1.76	<0.001
Age		0.020	0.00, 0.04	0.011	-0.005	-0.02, 0.01	0.364
BMI		0.002	-0.01, 0.01	0.766	0.004	-0.01, 0.01	0.480
Ethnicity**	Asian	0.250	-0.03, 0.53	0.076	0.280	0.06, 0.50	0.011
	Black	0.670	0.21, 1.12	0.004	0.720	0.36, 1.07	<0.001
	Mixed	0.180	-0.45, 0.80	0.577	0.310	-0.16, 0.79	0.194
	Other	0.850	0.39, 1.30	<0.001	0.480	0.13, 0.84	0.008

*Neither was used as the Study group reference category

**White was used as the Ethnicity reference category
