# Supplementary material for: Maternal and Placental Antibody Responses in SARS-CoV-2 Vaccination and Natural Infection During Pregnancy
Source: Pediatr Infect Dis J. 2025 Feb 14;44(2):S32–7. doi: 10.1097/INF.0000000000004704 (PMC7617455; doi:10.1097/INF.0000000000004704)
Supplement: Supplementary file 8 [file inf-44-s032-s008.pdf]

Supplemental Digital Content 8. Simple and multiple linear regression results for log-transformed maternal ADCD S assay results at delivery

Explanatory variables		Simple linear regression			Multiple linear regression		
		Estimate	95% CI	p value	Estimate	95% CI	p value
Study group	Infected	0.720	0.63, 0.81	<0.001	0.620	0.51, 0.73	<0.001
	Vaccinated	1.050	0.89, 1.21	<0.001	1.020	0.87, 1.17	<0.001
	Both	1.870	1.70, 2.04	<0.001	1.830	1.65, 2.00	<0.001
Age		0.020	0.01, 0.03	<0.001	0.004	0.00, 0.01	0.218
BMI		0.007	-0.01, 0.00	0.08	-0.002	-0.01, 0.00	0.488
Ethnicity	Asian	0.180	0.00, 0.35	0.045	0.100	-0.02, 0.22	0.105
	Black	-0.090	-0.37, 0.19	0.546	0.040	-0.16, 0.23	0.716
	Mixed	0.010	-0.37, 0.39	0.957	0.170	-0.09, 0.43	0.203
	Other	0.410	0.13, 0.69	0.004	0.200	0.01, 0.40	0.043

Supplemental Digital Content 9. Simple and multiple linear regression results for log-transformed maternal ADCD N assay results at delivery

Explanatory variables		Simple linear regression			Multiple linear regression		
		Estimate	95% CI	p value	Estimate	95% CI	p value
Study group	Infected	0.653	0.58, 0.72	<0.001	0.520	0.45, 0.60	<0.001
	Vaccinated	0.010	-0.11, 0.14	0.827	-0.001	-0.11, 0.11	0.991
	Both	0.530	0.40, 0.67	<0.001	0.560	0.43, 0.68	<0.001
Age		0.010	0.00, 0.02	0.002	0.001	0.00, 0.01	0.626
BMI		-0.001	-0.01, 0.00	0.589	0.001	0.00, 0.00	0.752
Ethnicity	Asian	0.080	-0.03, 0.19	0.138	0.110	0.02, 0.20	0.013
	Black	0.030	-0.15, 0.20	0.772	0.050	-0.09, 0.19	0.496
	Mixed	-0.120	-0.36, 0.11	0.308	-0.070	-0.26, 0.12	0.467
	Other	0.380	0.20, 0.55	<0.001	0.220	0.08, 0.36	0.003

Supplemental Digital Content 10. Simple and multiple linear regression results for log-transformed maternal Euroimmun assay results at delivery

Explanatory variables		Simple linear regression			Multiple linear regression		
		Estimate	95% CI	p value	Estimate	95% CI	p value
Study group	Infected	0.950	0.85, 1.05	<0.001	0.880	0.75, 1.01	<0.001
	Vaccinated	1.410	1.22, 1.59	<0.001	1.390	1.20, 1.58	<0.001
	Both	1.870	1.68, 2.07	<0.001	1.820	1.60, 2.03	<0.001
Age		0.020	0.01, 0.03	<0.001	0.001	-0.01, 0.01	0.868
BMI		-0.010	-0.02, 0.00	0.038	-0.004	-0.01, 0.00	0.295
Ethnicity	Asian	0.260	0.06, 0.47	0.011	0.150	0.00, 0.30	0.044
	Black	0.050	-0.28, 0.38	0.777	0.200	-0.04, 0.43	0.105
	Mixed	0.140	-0.31, 0.59	0.541	0.330	0.01, 0.65	0.046
	Other	0.500	0.17, 0.82	0.003	0.230	-0.01, 0.47	0.065

Supplemental Digital Content 11. Simple and multiple linear regression results for log-transformed maternal Roche S assay results at delivery

Explanatory variables		Simple linear regression			Multiple linear regression		
		Estimate	95% CI	p value	Estimate	95% CI	p value

Study group	Infected	1.620	1.42, 1.82	<0.001	1.630	1.37, 1.90	<0.001
	Vaccinated	2.260	1.90, 2.63	<0.001	2.250	1.87, 2.62	<0.001
	Both	3.540	3.15, 3.92	<0.001	3.510	3.07, 3.95	<0.001
Age		0.050	0.03, 0.06	<0.001	0.005	-0.01, 0.02	0.521
BMI		-0.010	-0.03, 0.01	0.177	-0.002	-0.02, 0.01	0.741
Ethnicity	Asian	0.470	0.08, 0.86	0.018	0.260	-0.03, 0.56	0.082
	Black	0.390	-0.24, 1.02	0.227	0.620	0.14, 1.10	0.011
	Mixed	0.280	-0.58, 1.15	0.518	0.610	-0.04, 1.25	0.066
	Other	0.960	0.32, 1.59	0.003	0.540	0.06, 1.03	0.029

*Neither was used as the Study group reference category

**White was used as the Ethnicity reference category
