# Supplementary material for: Maternal and Placental Antibody Responses in SARS-CoV-2 Vaccination and Natural Infection During Pregnancy
Source: Pediatr Infect Dis J. 2025 Feb 14;44(2):S32–7. doi: 10.1097/INF.0000000000004704 (PMC7617455; doi:10.1097/INF.0000000000004704)
Supplement: Supplementary file 9 [file inf-44-s032-s009.pdf]

SUPPLEMENTAL DIGITAL CONTENT 12. Transfer ratios from mother to cord at delivery amongst positive participants only (geometric mean)

		ADCD N	ADCD S	Roche N	Roche S	Euroimmun	MNA	ACDA2I	ADNP
All participants		1.1	1.3	1.1	0.8	1.1	0.7	1.2	1.2
By study group	Infected (n=132)	1.4	1.1	1.1	0.8	1.2	0.7	1.2	1.1
	Vaccinated (n=31)	-	1.1	-	0.7	0.9	-	1.3	1.3
	Both (n=27)	1.0	1.1	1.3	1.1	1.7	-	1.1	1.3
