# Supplementary material for: Maternal and Placental Antibody Responses in SARS-CoV-2 Vaccination and Natural Infection During Pregnancy
Source: Pediatr Infect Dis J. 2025 Feb 14;44(2):S32–7. doi: 10.1097/INF.0000000000004704 (PMC7617455; doi:10.1097/INF.0000000000004704)

SUPPLEMENTAL DDIGITAL CONTENT 13. Transfer ratios from mother to cord at delivery amongst COVID infected participants for ADCD S (n = 98), ADCD N (n = 95), ADNP (n = 57), ACDA2I (n = 77), Roche S (n = 113), Roche N (n = 99), Euroimmun (n = 69), MNA (n = 32). Results outside the reportable range of each assay were excluded. Results from pertussis SBA are from all treatment groups (n = 44). Geometric mean with 95% CI plotted.

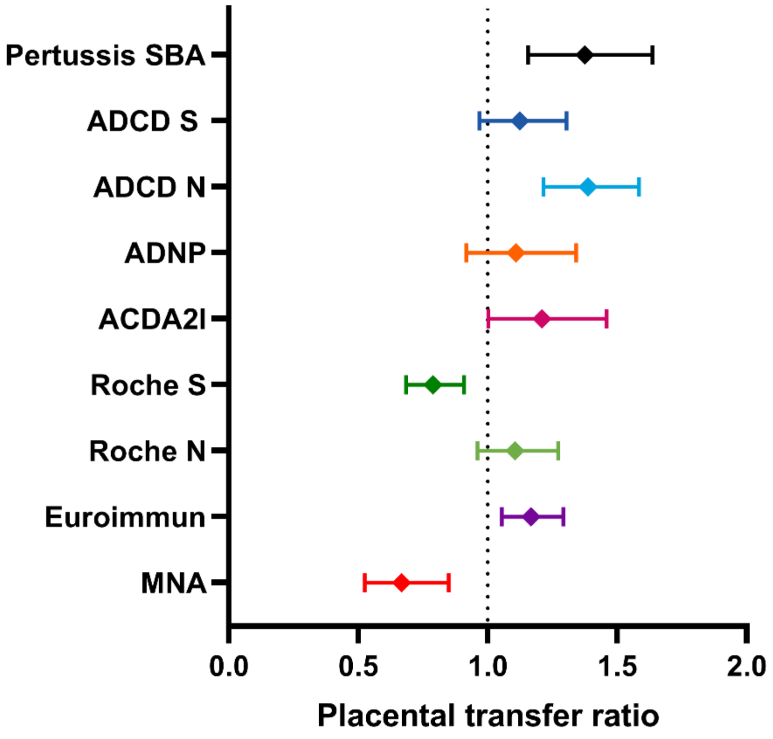

Supplement: Supplementary file 10 [file inf-44-s032-s010.pdf]
