# Supplementary material for: Maternal and Placental Antibody Responses in SARS-CoV-2 Vaccination and Natural Infection During Pregnancy
Source: Pediatr Infect Dis J. Author manuscript; Available in PMC 2025 Mar 4. (PMC7617455; doi:10.1097/INF.0000000000004704)
Supplement: Supplementary file 1 [file EMS202779-supplement-Edited_final_abstract.docx]

**Abstract** 

**Background** 

As COVID-19 becomes endemic, understanding antibody response and transfer during pregnancy is crucial to inform policy and vaccination schedules. Whilst good immunogenicity has been shown from SARS-CoV-2 vaccines, few data are available demonstrating functional responses in pregnant populations and infants. 

**Methods** 

A prospective multi-site observational study was completed across 14 centres in England from 23rd April 2020 to 21st December 2022. Demographic, COVID infection and vaccination data were collected. Maternal and cord blood samples were taken at delivery, with maternal and neonatal blood samples taken at 6 weeks for participants who had been infected or vaccinated. Antibody concentrations were measured using antibody-dependent complement deposition, antibody-dependent neutrophil phagocytosis, ACE2 inhibition, and Roche and EuroImmun antibody binding assays at UK Health Security Agency.

**Results**

Maternal vaccination and infection both produced an antibody response in 100% of mothers and 93.8% and 92.9% of neonates respectively, which persisted at 6 weeks in 95%. The strongest response was seen in mothers who were both vaccinated and infected. Anti-spike antibody response decreased almost 25-fold from first to third trimester vaccination (p=0.013). Placental transfer of antibodies post-infection showed varied results depending on the assay used, with higher transfer ratios observed in assays measuring Fc-mediated antibody effector functions and IgG-specific responses. 

**Conclusion**

Maternal vaccination is associated with good immunogenicity and successful antibody transfer to the neonate, particularly with vaccination in early pregnancy. Further study is needed to determine the mechanism by which timing of vaccination affects antibody transfer. When measuring placental transfer of antibodies, consideration of the assay to use is essential.
