# Supplementary material for: Maternal and Placental Antibody Responses in SARS-CoV-2 Vaccination and Natural Infection During Pregnancy
Source: Pediatr Infect Dis J. Author manuscript; Available in PMC 2025 Mar 4. (PMC7617455; doi:10.1097/INF.0000000000004704)
Supplement: Supplementary file 2 [file EMS202779-supplement-SDC_1.docx]

**SUPPLEMENTAL DIGITAL CONTENT 1.** Methods

*Antibody-dependent complement deposition (ADCD) assay* 

SPHERO carboxyl magnetic blue-fluorescent beads of varying intensities (Spherotech, USA) were coupled with SARS-CoV-2 whole spike protein (Lake Pharma, 46328) and recombinant nucleoprotein prepared in-house using a two-step sulpho-NHS/EDC process44. To prepare the nucleoprotein, the full-length SARS-CoV-2 nucleocapsid gene was optimised for expression in human cells and synthesised and subcloned into pcDNA3.1 expression vector (GeneArt, ThermoFisher) for transient expression in Expi293 cells. The nucleoprotein was expressed with a C-terminal His(6) tag and purified from the cytosolic fraction after detergent lysis and sonication using a 5 ml HisTrap excel column (as per manufacturer’s instructions,  Cytiva). The eluted pool was dialysed against 50 mM HEPES, NaOH, pH8.0, before being applied to a 5 ml SP-Sepharose HiTrap column. Following elution with 50 mM HEPES, NaOH, pH8.0 + 1M NaCl (gradient elution, 20 column volumes) purified nucleoprotein was dialysed against 50 mM HEPES-NaOH, pH8.0, 250 mM NaCl, 1 mM EDTA, 20% (w/v) sucrose and stored at -80^o^C. Heat-inactivated NIBSC Anti-SARS-CoV-2 Antibody Diagnostic Calibrant (NIBSC, 20/162) at an initial 1:40 dilution in blocking buffer (BB; PBS, 2% BSA) was added in duplicate to a V-bottom 96-well microtiter plate and serially diluted 2:3 in 20 µL BB. Heat-inactivated test serum (3 µL in duplicate) were added to 27 µL BB and serially diluted 1:3 in 20 µL BB. This was followed by 20 µL of SARS-CoV-2 spike and nucleocapsid protein-coated magnetic beads (50 beads per µL for each bead type) to give a multiplexed assay with a final 1:3 serial dilution range starting at 1:20. The serial dilution for NIBSC 20/162 standard started at 1:80. The mixture was incubated at 25°C for 30 min with shaking (900rpm). The beads were washed twice in 200 µL wash buffer (WB; BB+0.05% Tween-20), then resuspended in 50µL BB containing 12.5% IgG- and IgM-depleted human plasma45 and incubated at 37°C for 15 min with shaking (900rpm). Beads were next washed twice with 200 µL WB and resuspended in 100 µL FITC-conjugated rabbit anti-human C3c polyclonal antibody (Abcam) diluted 1:500 in BB and incubated in the dark at 25°C for 20 min. After two more washes with 200 µL WB, the samples were resuspended in 40 µL HBSS and analysed using an iQue Screener Plus® with iQue Forecyt® Standard Edition 9.0 (R3) software (Sartorius, Germany). For each sample, a minimum of 50 beads (per bead type) were collected. Conjugated beads were gated based on forward scatter and side scatter and then spike-conjugated and nucleocapsid-conjugated beads separated and gated by fluorescence in the BL4 channel (488 nm – 675/30 nm). The fluorescent-bead population was gated and measured for FITC Median Fluorescent Intensity (MFI), which represents deposition of C3b/iC3b. The NIBSC 20/162 calibrant was assigned an arbitrarily unitage of 1,000 complement activating units (CAU)/mL and plotted as a 4PL curve with 1/Y2 weighting and the linear range calculated. The mean MFI from each sample was interpolated against the NIBSC 20/162 4PL curve and based on the concentration that hit the linear range was multiplied by the dilution factor to assign activity of the sera as CAU.

*Antibody-dependent neutrophil phagocytosis (ADNP) assay* 

The ADNP protocol was adapted from previous studies46.  1 µm carboxylate-modified crimson FluoSpheres™ beads (Thermo Fisher, USA) were coupled with SARS-CoV-2 whole spike protein (Lake Pharma, 46328) using a two-step sulpho-NHS/EDC process ^25^. Heat-inactivated NIBSC Anti-SARS-CoV-2 Antibody Diagnostic Calibrant (NIBSC, 20/162) at an initial 1:85 dilution in DPBS-GACM buffer (Dulbecco’s PBS supplemented with 5.5 mM glucose, 0.5% w/v BSA, 0.9 mM CaCl2 and 0.5 mM MgSO4 at pH 7.4) was added to a V-bottom 96-well microtiter plate and serially diluted 5:6 in 20 µL DPBS-GACM. Heat-inactivated test sera (5 µL) were added to 20 µL DPBS-GACM and serially diluted 1:2 in 12 µL DPBS-GACM. 5 μL from the calibrant and the test sera serial dilutions were transferred in duplicate to a U-bottom 96-well microtiter plate, followed by the addition of 5 μL spike-conjugated beads (4×10^4^ beads/μL). The serial dilution for NIBSC 20/162 standard started at 1:170, and for test samples started at 1:10. The mixture was incubated at 37°C for 1h with shaking (900rpm). Next, 5 μL of IgG- and IgM-depleted human plasma45 diluted 1/10 in DPBS-GACM was added to the mix as an exogenous complement source and this was immediately followed by the addition of 10 μL of granulocyte-differentiated HL-60 cells (ATTC, CCL-240; differentiated with 0.8% N,N-dimethylformamide for 5 days) resuspended in DPBS-GACM at a concentration of 2×10^6^ cells/mL. The plate was incubated at 37°C for a further 1h with shaking (900rpm). At the end, phagocytosis was stopped by placing the plate on ice and adding 20μL of cold DPBS with 0.02% w/v EDTA in every well. Samples were analysed using an iQue Screener Plus® with iQue Forecyt® Standard Edition 9.0 (R3) software (Sartorius, Germany). Phagocytising cells were gated based on forward scatter and side scatter and MFI in the BL4 channel (488 nm – 675/30 nm) measured, which represents uptake of fluorescent beads. The NIBSC 20/162 calibrant was arbitrarily designated an opsonophagocytic activity equal to 1,000 phagocytosis units (PU)/mL and plotted as a 4PL curve with 1/Y2 weighting and the linear range calculated. The mean MFI from each sample was interpolated against the NIBSC 20/162 4PL curve and based on the concentration that hit the linear range was multiplied by the dilution factor to assign activity of the sera as Interpolated Phagocytosis Units (IPU).

*Antibody and complement-dependent ACE2 inhibition (ACDA2I) assay* 

SPHERO carboxyl magnetic blue-fluorescent beads were coupled with SARS-CoV-2 whole spike protein as above. Heat-inactivated NIBSC 20/162 at an initial 1:25 dilution in BB was added in duplicate to a V-bottom 96-well microtiter plate and serially diluted 2:3 in 20 µL BB. Heat-inactivated test serum (12 µL in duplicate) were added to 48µL BB and serially diluted 2:3 in 20 µL BB. This was followed by 20 µL of SARS-CoV-2 spike protein-coated magnetic beads (50 beads per µL) to give a final 2:3 serial dilution range starting at 1:10. The serial dilution for NIBSC 20/162 standard started at 1:50. The mixture was incubated at 25°C for 30 min with shaking (900rpm). The beads were washed twice in 200µL wash buffer (WB; BB+0.05% Tween-20), then resuspended in 50 µL BB containing 10% IgG- and IgM-depleted human plasma45 and incubated at 37°C for 15 min with shaking (900rpm). Beads were next washed twice with 200 µL WB and resuspended in 50 µL Human ACE2 (18-615) Recombinant Protein, Sheep FC-Tag (The Native Antigen Company Limited, REC31876) at 1.0 µg/mL concentration in BB and incubated at 37°C for 30 min with shaking (900rpm). After two more washes with 200 µL WB, the samples were resuspended in 100 µL FITC-conjugated AffiniPure Rabbit Anti-Sheep Polyclonal IgG, Fc-Fragment Specific (Jackson ImmunoResearch) at 1:500 dilution for 20 min in the dark at 25°C with shaking (400rpm). Following two final washes with 200 µL WB, the samples were resuspended in 20 µL HBSS and analysed using an iQue Screener Plus® with iQue Forecyt® Standard Edition 9.0 (R3) software as above. The fluorescent-bead population was gated and measured for FITC MFI, which quantifies inhibition of Spike-ACE2 interaction due to the formation of antibody-complement immune complexes. The NIBSC 20/162 calibrant was assigned an arbitrarily unitage of 1,000 ACE-2 inhibitory units (AIU)/mL and plotted as a 4PL curve with 1/Y2 weighting and the linear range calculated.  The mean MFI from each sample was interpolated against the NIBSC 20/162 4PL curve and based on the concentration that hit the linear range was multiplied by the dilution factor to assign activity of the sera as AIU.

*Antibody binding*

SARS-CoV-2 antibody testing was performed using commercial platforms. Roche Elecsys Anti-SARS-CoV-2 S (Roche S) and Anti-SARS-CoV-2 (Roche N) electrochemiluminescence immunoassay (ECLIA) which measures total antibodies to the spike protein RBD or nucleocapsid protein47,48. As well as EuroImmun Anti-SARS-CoV-2 QuantiVac enzyme-linked immunosorbent assay (ELISA) (IgG) targeting the S1 antigen^28^. While the Roche S and EuroImmun assays are either quantitative or semi-quantitative, reporting results as units (U)/mL and relative units (RU)/mL respectively, the Roche N assay is primarily qualitative. Nonetheless, the signal/cut-off (S/CO) ratio used in the Roche N assay provides a relative measure of antibody levels within the sample by comparing the signal generated by the sample to that of an internal reactive calibrator.

*Microneutralization assay (MNA)*

MNAs were performed on live virus SARS-CoV-2/human/AUS/VIC01/2020 [Victoria] strain and neutralisation titres giving 50% focus reduction determined 49.
