# Supplementary material for: Maternal and Placental Antibody Responses in SARS-CoV-2 Vaccination and Natural Infection During Pregnancy
Source: Pediatr Infect Dis J. Author manuscript; Available in PMC 2025 Mar 4. (PMC7617455; doi:10.1097/INF.0000000000004704)
Supplement: Supplementary file 3 [file EMS202779-supplement-SDC_12.docx]

**SUPPLEMENTAL DIGITAL CONTENT 12.** Transfer ratios from mother to cord at delivery amongst positive participants only (geometric mean)

|  |  | ADCD N | ADCD S | Roche N | Roche S | Euroimmun | MNA | ACDA2I | ADNP |
| --- | --- | --- | --- | --- | --- | --- | --- | --- | --- |
| All participants |  | 1.1 | 1.3 | 1.1 | 0.8 | 1.1 | 0.7 | 1.2 | 1.2 |
| By study group | Infected (n=132) | 1.4 | 1.1 | 1.1 | 0.8 | 1.2 | 0.7 | 1.2 | 1.1 |
|  | Vaccinated (n=31) | - | 1.1 | - | 0.7 | 0.9 | - | 1.3 | 1.3 |
|  | Both (n=27) | 1.0 | 1.1 | 1.3 | 1.1 | 1.7 | - | 1.1 | 1.3 |
