# Supplementary material for: Maternal and Placental Antibody Responses in SARS-CoV-2 Vaccination and Natural Infection During Pregnancy
Source: Pediatr Infect Dis J. Author manuscript; Available in PMC 2025 Mar 4. (PMC7617455; doi:10.1097/INF.0000000000004704)
Supplement: Supplementary file 4 [file EMS202779-supplement-SDC_13.docx]

**SUPPLEMENTAL DDIGITAL CONTENT 13.** Transfer ratios from mother to cord at delivery amongst COVID infected participants for ADCD S (n = 98), ADCD N (n = 95), ADNP (n = 57), ACDA2I (n = 77), Roche S (n = 113), Roche N (n = 99), Euroimmun (n = 69), MNA (n = 32). Results outside the reportable range of each assay were excluded. Results from pertussis SBA are from all treatment groups (n = 44). Geometric mean with 95% CI plotted.
