# Supplementary material for: Maternal and Placental Antibody Responses in SARS-CoV-2 Vaccination and Natural Infection During Pregnancy
Source: Pediatr Infect Dis J. Author manuscript; Available in PMC 2025 Mar 4. (PMC7617455; doi:10.1097/INF.0000000000004704)
Supplement: Supplementary file 5 [file EMS202779-supplement-SDC_2.docx]

**SUPPLEMENTAL DIGITAL CONTENT 2.** Characteristics of participants

|  |  | All (n=881) | Infected (n=173) | Vaccinated (n=43) | Both (n=38) | Neither (n=627) |
| --- | --- | --- | --- | --- | --- | --- |
| Age (years) | Median | 32.0 | 33.0 | 34.0 | 33.5 | 31.0 |
|  | IQR | 28.0-32.0 | 29.0-36.0 | 30.5-37.0 | 31.3-36.8 | 27.0-35.0 |
| BMI (kg/m^2^) | Median | 27.0 | 27.0 | 24.5 | 25.6 | 27.0 |
|  | IQR | 23.0-31.6 | 23.0-33.1 | 23.0-27.0 | 23.0-28.2 | 23.0-31.7 |
| Ethnicity N (%) | White | 637 (72%) | 85 (49%) | 32 (74%) | 24 (63%) | 496 (79%) |
|  | Asian/Asian British | 75 (9%) | 9 (5%) | 8 (19%) | 4 (11%) | 54 (9%) |
|  | Black/African/Caribbean/British | 24 (3%) | 4 (2%) | 0 (0%) | 0 (0%) | 20 (3%) |
|  | Other | 23 (3%) | 8 (5%) | 2 (5%) | 1 (3%) | 12 (2%) |
|  | Mixed/Multiple | 13 (1%) | 1 (1%) | 0 (0%) | 0 (0%) | 12 (2%) |
|  | Not stated | 109 (12%) | 66 (38%) | 1 (2%) | 9 (24%) | 33 (5%) |
