# Supplementary material for: Maternal and Placental Antibody Responses in SARS-CoV-2 Vaccination and Natural Infection During Pregnancy
Source: Pediatr Infect Dis J. Author manuscript; Available in PMC 2025 Mar 4. (PMC7617455; doi:10.1097/INF.0000000000004704)
Supplement: Supplementary file 7 [file EMS202779-supplement-SDC_4.docx]

**SUPPLEMENTAL DIGITAL CONTENT 4.**  Maternal antibody results at delivery (geometric mean) 

|  |  | Maternal antibody results at delivery |  |  |  |  |
| --- | --- | --- | --- | --- | --- | --- |
|  |  | ADCD N (CAU/mL) | ADCD S (CAU/mL) | Roche N (S/CO ratio) | Roche S (U/mL) | Euroimmun (RU/mL) |
| Study group | Infected | 82.2 | 37.2 | 12.5 | 62.2 | 1.7 |
|  | Vaccinated | 18.9 | 80.3 | 0.1 | 278.4 | 4.8 |
|  | Both | 61.0 | 590.5 | 3.9 | 6523.5 | 15.5 |
|  | Neither | 18.2 | 7.2 | 0.2 | 1.5 | 0.2 |
|  | p value* | <0.001 | <0.001 | <0.001 | <0.001 | <0.001 |
| Trimester of infection | First | 33.0 | 42.2 | 8.9 | 221.2 | 2.0 |
|  | Second | 60.8 | 45.1 | 13.7 | 127.3 | 1.7 |
|  | Third | 145.9 | 125.8 | 11.0 | 276.6 | 2.4 |
|  | p value* | 0.001 | 0.024 | 0.734 | 0.372 | 0.041 |
| Trimester of vaccination | First | 43.4 | 349.3 | 3.7 | 5005.6 | 3.1 |
|  | Second | 27.9 | 262.3 | 0.3 | 3284.6 | 11.1 |
|  | Third | 30.5 | 95.3 | 0.4 | 207.1 | 4.2 |
|  | p value* | 0.129 | 0.055 | 0.155 | 0.013 | 0.107 |

*p value for Kruskal Wallis test
