# Supplementary material for: Maternal and Placental Antibody Responses in SARS-CoV-2 Vaccination and Natural Infection During Pregnancy
Source: Pediatr Infect Dis J. Author manuscript; Available in PMC 2025 Mar 4. (PMC7617455; doi:10.1097/INF.0000000000004704)
Supplement: Supplementary file 8 [file EMS202779-supplement-SDC_5.docx]

**SUPPLEMENTAL DIGITAL CONTENT** **5**.  Neonatal antibody results at delivery (cord) for a) antibody-dependent complement deposition assay against spike protein, b) antibody-dependent complement deposition assay against nucleocapsid protein, c) antibody-dependant neutrophil phagocytosis, d) antibody and complement-dependant ACE2 inhibition assay, e) Roche total antibody binding to RBD, f) Roche total antibody binding to nucleocapsid protein, g) Euroimmun IgG binding to S1 antigen. Geometric mean with 95% CI plotted.
