# Supplementary material for: Maternal and Placental Antibody Responses in SARS-CoV-2 Vaccination and Natural Infection During Pregnancy
Source: Pediatr Infect Dis J. Author manuscript; Available in PMC 2025 Mar 4. (PMC7617455; doi:10.1097/INF.0000000000004704)
Supplement: Supplementary file 9 [file EMS202779-supplement-SDC_6.docx]

**SUPPLEMENTAL DIGITAL CONTENT**  **6**. Neonatal antibody results at delivery (geometric mean)

|  |  | ADCD N (CAU/mL) | ADCD S (CAU/mL) | Roche N (S/CO ratio) | Roche S (U/mL) | Euroimmun (RU/mL) |
| --- | --- | --- | --- | --- | --- | --- |
| Study group | Infected | 89.0 | 35.9 | 9.9 | 39.0 | 1.7 |
|  | Vaccinated | 18.3 | 83.9 | 0.1 | 203.8 | 3.3 |
|  | Both | 64.6 | 549.0 | 3.5 | 5831.8 | 12.4 |
|  | Neither | 17.1 | 7.5 | 0.2 | 1.5 | 0.2 |
|  | p value* | <0.001 | <0.001 | <0.001 | <0.001 | <0.001 |
| Trimester of infection | First | 28.5 | 57.9 | 12.8 | 270.3 | 1.9 |
|  | Second | 75.0 | 54.7 | 18.2 | 103.5 | 2.3 |
|  | Third | 124.3 | 88.8 | 5.4 | 128.4 | 1.9 |
|  | p value* | 0.001 | 0.470 | 0.217 | 0.269 | 0.464 |
| Trimester of vaccination | First | 58.9 | 387.5 | 4.3 | 6873.9 | 5.0 |
|  | Second | 24.9 | 343.5 | 0.4 | 8775.0 | 11.7 |
|  | Third | 26.8 | 62.6 | 0.4 | 89.7 | 3.6 |
|  | p value* | 0.565 | 0.006 | 0.645 | 0.002 | 0.044 |

*p value for Kruskal Wallis test
