# Supplementary material for: Maternal and Placental Antibody Responses in SARS-CoV-2 Vaccination and Natural Infection During Pregnancy
Source: Pediatr Infect Dis J. Author manuscript; Available in PMC 2025 Mar 4. (PMC7617455; doi:10.1097/INF.0000000000004704)
Supplement: Supplementary file 10 [file EMS202779-supplement-SDC_7.docx]

**SUPPLEMENTAL DIGITAL CONTENT 7.** Simple and multiple linear regression results for log-transformed maternal Roche N assay results at delivery

| Explanatory variables |  | Simple linear regression |  |  | Multiple linear regression |  |  |
| --- | --- | --- | --- | --- | --- | --- | --- |
|  |  | Estimate | 95% CI | p value | Estimate | 95% CI | p value |
| Study group* | Infected | 1.850 | 1.69, 2.00 | <0.001 | 1.840 | 1.65, 2.04 | <0.001 |
|  | Vaccinated | -0.180 | -0.46, 0.10 | 0.204 | -0.170 | -0.45, 0.10 | 0.219 |
|  | Both | 1.380 | 1.09, 1.68 | <0.001 | 1.440 | 1.12, 1.76 | <0.001 |
| Age |  | 0.020 | 0.00, 0.04 | 0.011 | -0.005 | -0.02, 0.01 | 0.364 |
| BMI |  | 0.002 | -0.01, 0.01 | 0.766 | 0.004 | -0.01, 0.01 | 0.480 |
| Ethnicity** | Asian | 0.250 | -0.03, 0.53 | 0.076 | 0.280 | 0.06, 0.50 | 0.011 |
|  | Black | 0.670 | 0.21, 1.12 | 0.004 | 0.720 | 0.36, 1.07 | <0.001 |
|  | Mixed | 0.180 | -0.45, 0.80 | 0.577 | 0.310 | -0.16, 0.79 | 0.194 |
|  | Other | 0.850 | 0.39, 1.30 | <0.001 | 0.480 | 0.13, 0.84 | 0.008 |

*Neither was used as the Study group reference category

**White was used as the Ethnicity reference category
