# Supplementary material for: Maternal and Placental Antibody Responses in SARS-CoV-2 Vaccination and Natural Infection During Pregnancy
Source: Pediatr Infect Dis J. Author manuscript; Available in PMC 2025 Mar 4. (PMC7617455; doi:10.1097/INF.0000000000004704)
Supplement: Supplementary file 11 [file EMS202779-supplement-SDC_8_11.docx]

**Supplemental Digital Content 8.** Simple and multiple linear regression results for log-transformed maternal ADCD S assay results at delivery

| Explanatory variables |  | Simple linear regression |  |  | Multiple linear regression |  |  |
| --- | --- | --- | --- | --- | --- | --- | --- |
|  |  | Estimate | 95% CI | p value | Estimate | 95% CI | p value |
| Study group | Infected | 0.720 | 0.63, 0.81 | <0.001 | 0.620 | 0.51, 0.73 | <0.001 |
|  | Vaccinated | 1.050 | 0.89, 1.21 | <0.001 | 1.020 | 0.87, 1.17 | <0.001 |
|  | Both | 1.870 | 1.70, 2.04 | <0.001 | 1.830 | 1.65, 2.00 | <0.001 |
| Age |  | 0.020 | 0.01, 0.03 | <0.001 | 0.004 | 0.00, 0.01 | 0.218 |
| BMI |  | 0.007 | -0.01, 0.00 | 0.08 | -0.002 | -0.01, 0.00 | 0.488 |
| Ethnicity | Asian | 0.180 | 0.00, 0.35 | 0.045 | 0.100 | -0.02, 0.22 | 0.105 |
|  | Black | -0.090 | -0.37, 0.19 | 0.546 | 0.040 | -0.16, 0.23 | 0.716 |
|  | Mixed | 0.010 | -0.37, 0.39 | 0.957 | 0.170 | -0.09, 0.43 | 0.203 |
|  | Other | 0.410 | 0.13, 0.69 | 0.004 | 0.200 | 0.01, 0.40 | 0.043 |

**Supplemental Digital Content 9.** Simple and multiple linear regression results for log-transformed maternal ADCD N assay results at delivery

| Explanatory variables |  | Simple linear regression |  |  | Multiple linear regression |  |  |
| --- | --- | --- | --- | --- | --- | --- | --- |
|  |  | Estimate | 95% CI | p value | Estimate | 95% CI | p value |
| Study group | Infected | 0.653 | 0.58, 0.72 | <0.001 | 0.520 | 0.45, 0.60 | <0.001 |
|  | Vaccinated | 0.010 | -0.11, 0.14 | 0.827 | -0.001 | -0.11, 0.11 | 0.991 |
|  | Both | 0.530 | 0.40, 0.67 | <0.001 | 0.560 | 0.43, 0.68 | <0.001 |
| Age |  | 0.010 | 0.00, 0.02 | 0.002 | 0.001 | 0.00, 0.01 | 0.626 |
| BMI |  | -0.001 | -0.01, 0.00 | 0.589 | 0.001 | 0.00, 0.00 | 0.752 |
| Ethnicity | Asian | 0.080 | -0.03, 0.19 | 0.138 | 0.110 | 0.02, 0.20 | 0.013 |
|  | Black | 0.030 | -0.15, 0.20 | 0.772 | 0.050 | -0.09, 0.19 | 0.496 |
|  | Mixed | -0.120 | -0.36, 0.11 | 0.308 | -0.070 | -0.26, 0.12 | 0.467 |
|  | Other | 0.380 | 0.20, 0.55 | <0.001 | 0.220 | 0.08, 0.36 | 0.003 |

**Supplemental Digital Content 10.** Simple and multiple linear regression results for log-transformed maternal Euroimmun assay results at delivery

| Explanatory variables |  | Simple linear regression |  |  | Multiple linear regression |  |  |
| --- | --- | --- | --- | --- | --- | --- | --- |
|  |  | Estimate | 95% CI | p value | Estimate | 95% CI | p value |
| Study group | Infected | 0.950 | 0.85, 1.05 | <0.001 | 0.880 | 0.75, 1.01 | <0.001 |
|  | Vaccinated | 1.410 | 1.22, 1.59 | <0.001 | 1.390 | 1.20, 1.58 | <0.001 |
|  | Both | 1.870 | 1.68, 2.07 | <0.001 | 1.820 | 1.60, 2.03 | <0.001 |
| Age |  | 0.020 | 0.01, 0.03 | <0.001 | 0.001 | -0.01, 0.01 | 0.868 |
| BMI |  | -0.010 | -0.02, 0.00 | 0.038 | -0.004 | -0.01, 0.00 | 0.295 |
| Ethnicity | Asian | 0.260 | 0.06, 0.47 | 0.011 | 0.150 | 0.00, 0.30 | 0.044 |
|  | Black | 0.050 | -0.28, 0.38 | 0.777 | 0.200 | -0.04, 0.43 | 0.105 |
|  | Mixed | 0.140 | -0.31, 0.59 | 0.541 | 0.330 | 0.01, 0.65 | 0.046 |
|  | Other | 0.500 | 0.17, 0.82 | 0.003 | 0.230 | -0.01, 0.47 | 0.065 |

**Supplemental Digital Content 11****.** Simple and multiple linear regression results for log-transformed maternal Roche S assay results at delivery

| Explanatory variables |  | Simple linear regression |  |  | Multiple linear regression |  |  |
| --- | --- | --- | --- | --- | --- | --- | --- |
|  |  | Estimate | 95% CI | p value | Estimate | 95% CI | p value |
| Study group | Infected | 1.620 | 1.42, 1.82 | <0.001 | 1.630 | 1.37, 1.90 | <0.001 |
|  | Vaccinated | 2.260 | 1.90, 2.63 | <0.001 | 2.250 | 1.87, 2.62 | <0.001 |
|  | Both | 3.540 | 3.15, 3.92 | <0.001 | 3.510 | 3.07, 3.95 | <0.001 |
| Age |  | 0.050 | 0.03, 0.06 | <0.001 | 0.005 | -0.01, 0.02 | 0.521 |
| BMI |  | -0.010 | -0.03, 0.01 | 0.177 | -0.002 | -0.02, 0.01 | 0.741 |
| Ethnicity | Asian | 0.470 | 0.08, 0.86 | 0.018 | 0.260 | -0.03, 0.56 | 0.082 |
|  | Black | 0.390 | -0.24, 1.02 | 0.227 | 0.620 | 0.14, 1.10 | 0.011 |
|  | Mixed | 0.280 | -0.58, 1.15 | 0.518 | 0.610 | -0.04, 1.25 | 0.066 |
|  | Other | 0.960 | 0.32, 1.59 | 0.003 | 0.540 | 0.06, 1.03 | 0.029 |

*Neither was used as the Study group reference category

**White was used as the Ethnicity reference category
